# Simultaneous Moisture Content and Mass Flow Measurements in Wood Chip Flows Using Coupled Dielectric and Impact Sensors

**DOI:** 10.3390/s17010020

**Published:** 2016-12-23

**Authors:** Pengmin Pan, Timothy McDonald, John Fulton, Brian Via, John Hung

**Affiliations:** 1 Biosystems Engineering Department, Auburn University, 200 Corley Building, Auburn University, Auburn, AL 36849, USA; mcdontp@auburn.edu; 2 Department of Food, Agricultural and Biological Engineering, Ohio State University, 590 Woody Hayes Drive, Columbus, OH 43210-1057, USA; fulton.20@osu.edu; 3 School of Forestry and Wildlife Sciences, Auburn University, 602 Duncan Drive, Auburn University, Auburn, AL 36849, USA; bkv0003@auburn.edu; 4 Electrical & Computer Engineering Department, Auburn University, 200 Broun Hall, Auburn University, Auburn, AL 36849, USA; hungjoh@auburn.edu

**Keywords:** moisture content, biomass, electrical capacitance tomography, capacitance, mass flow

## Abstract

An 8-electrode capacitance tomography (ECT) sensor was built and used to measure moisture content (MC) and mass flow of pine chip flows. The device was capable of directly measuring total water quantity in a sample but was sensitive to both dry matter and moisture, and therefore required a second measurement of mass flow to calculate MC. Two means of calculating the mass flow were used: the first being an impact sensor to measure total mass flow, and the second a volumetric approach based on measuring total area occupied by wood in images generated using the capacitance sensor’s tomographic mode. Tests were made on 109 groups of wood chips ranging in moisture content from 14% to 120% (dry basis) and wet weight of 280 to 1100 g. Sixty groups were randomly selected as a calibration set, and the remaining were used for validation of the sensor’s performance. For the combined capacitance/force transducer system, root mean square errors of prediction (RMSEP) for wet mass flow and moisture content were 13.42% and 16.61%, respectively. RMSEP using the combined volumetric mass flow/capacitance sensor for dry mass flow and moisture content were 22.89% and 24.16%, respectively. Either of the approaches was concluded to be feasible for prediction of moisture content in pine chip flows, but combining the impact and capacitance sensors was easier to implement. In situations where flows could not be impeded, however, the tomographic approach would likely be more useful.

## 1. Introduction

Wood chips are used as the raw material in pulp and paper production and as a feedstock for energy products from biomass. In both instances, the moisture content (MC) of the feedstock is an important concern. In kraft pulping, for example, MC affects the concentration of chemicals used in digestion of lignin, and in thermo-chemical energy production systems, moisture is typically considered a contaminant. Because of its effect on process control and costs, the value of the raw chipped material is therefore related to its MC, and a sensor to measure it can help in both determining value of feedstocks and in real-time control of conversion processes. A typical pulping facility in the southeastern US will consume many thousands of tonnes of chips daily so a process control moisture sensor in that scenario would have to be designed with a large capacity in mind, and preferably would not interfere with material handling systems already in place.

Examples of moisture sensors for woody materials abound in the literature and employ a variety of sensing technologies, including, for example, electrical resistance [1,2], acoustics [3,4], microwaves [5,6], and near infrared spectroscopy (NIR) [7,8]. Dielectric sensors have also been applied successfully using, for example, radio frequency spectroscopy [9], time, or frequency domain reflectometry [10,11], and direct capacitance measurements [12,13,14]. Each approach has its own merits, but most are not entirely practical for flowing streams of material as would be required in industrial scale process control applications.

A multi-electrode capacitance measurement system can be configured to operate in a tomographic mode, allowing the two-dimensional imaging of permittivity distribution [15]. With this capability, commonly referred to as electrical capacitance tomography, or ECT, a sensor can be built to determine, for example, the internal moisture content distribution of sawn lumber [16]. It has also been used to image physical properties of flowing streams of material and has been applied in determining void fraction in oil/gas mixtures [17], and in determining the mass flow rate of solid materials [18,19,20]. These systems have high capacity, are non-contact, and can be designed to operate practically in industrial process control applications.

Because it is sensitive to both dry matter and moisture phases, an ECT sensor should provide an ideal means of determining MC in wood chip flows in pulp and paper and energy applications. The main limitation of the system, however, is that the relative MC is not uniquely determined from permittivity measurements alone—it is also mass dependent. It is necessary, therefore, to decouple these two effects using domain-specific knowledge of the flow characteristics, as in [21], or use independent mass and moisture flow measurements to implement a real-time MC meter, which was the approach taken in this study.

The MC sensor developed for this project implemented two approaches for resolving the mass/moisture flow interaction. The first approach measured total mass flow and total moisture using independent impact and dielectric sensors, respectively. The second system used a single dielectric sensor in an ECT mode to simultaneously measure volumetric flow rate and total moisture content. The objective in this work was to characterize the relative accuracy of the two approaches and compare them from a practical standpoint as a means of determining MC of pine chip flows in industrial applications.

## 2. Experimental Section

### 2.1. Biomass Samples

**Figure 1:**
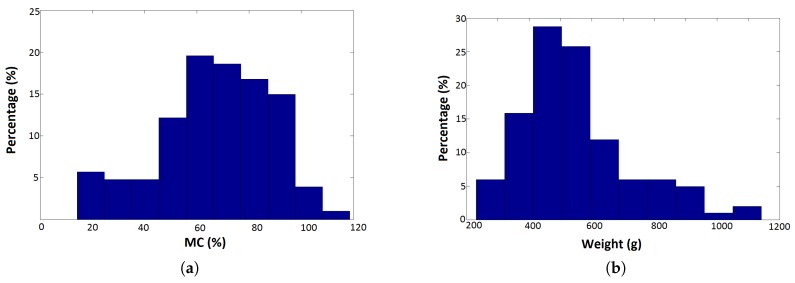
Distribution of sample numbers (**a**) by moisture content; and (**b**) by total (wet) weight.

Loblolly pine pulp-type wood chips were collected from three local sources near Auburn, AL, USA. Most were green chips collected from piles at industrial sites, but a portion of the samples had been air-dried for six months. All samples were stored in a $-20{}^{\circ }$C freezer when not being tested. The green chips were divided roughly equally into smaller portions and were dried in a 105 ${}^{\circ }$C oven for varying lengths of time to develop a range of MC. A total of 109 wood chip groups were obtained, nine of which had been derived from the initially air-dried chips. These groups were the experimental units on which all subsequent tests were run. Their range of moisture contents was from about 14.0% to 120.0% and wet weights were between 267.6 g and 1084.3 g. Distributions of sample MC and weights were plotted in Figure 1.

### 2.2. Experimental Methods

As pointed out in the Introduction, the output magnitude of a capacitive sensor is a composite of signals due to the quantities of both biomass and water under test. Without some independent knowledge of mass, or other (perhaps frequency response) information, moisture content cannot be estimated using the sensor alone.

In previous work, Pan et al. [22] developed an 8-electrode capacitance sensor for predicting MC of biomass and characterized its accuracy relative to an established estimation method (NIR). The sensor included a switching network to control the connection of electrodes which were activated in sequence and only one pair of electrodes (as a single capacitor) was coupled into the sensor at any moment. For the 8-electrode system, the total number of unique combinations of electrodes was 28 (eight choose two), and each combination provided an independent measure of the permittivity between them.

In the original application of the sensor, the paired capacitances from all electrode combinations were summed and related to the wet and dry weights of a sample, as in the following:

(1) $$\sum_{i=1}^{8}\sum_{j=i+1}^{8}\;C_{ij}=a+bM+cD,$$

where the terms *a*, *b*, and *c* were regression coefficients, *M* and *D* were total (wet) and dry weights of a single biomass sample, and $C_{ij}$ was measured capacitance between and electrodes *i* and *j*.

The model worked well in predicting dry weight of samples when total weight *M* was known (root mean squared error of prediction = 14.0%), and there was no interaction effect on the sensor output between the quantities of the two material phases. An independent total weight *M* was easily obtained in that study for the small, fixed samples tested and the equation could be used to predict dry weight and MC satisfactorily.

This relationship was also considered valid for the case of a moving biomass sample and was applied in the current study, although absolute weights were replaced by mass flow rates, as in the following. For a short time period Δt<<1 s equivalent to the sampling rate of the capacitance sensor, the mass flow rate of the material over that interval could be considered uniform and the sensor output *C* was linearly related to the flow rates of its two constituents, water (${\dot{m}}_{W}$) and dry wood (${\dot{m}}_{D}$):

(2) $$\sum_{i=1}^{8}\sum_{j=i+1}^{8}\;C_{ij}=C=\alpha +\beta {\dot{m}}_{W}+\gamma {\dot{m}}_{D},$$

where *α*, *β*, and *γ* were regression coefficients.

For the purposes of these tests, the mass flow rates could not be experimentally controlled and the above equation was not applied directly. Instead, an experiment consisted of sending a known, variable quantity of wood chips through the sensor over a fixed period of time, *T*, in this case, 5 s. The assumption was made that the cumulative predicted mass flow rates over the sampling period would be representative of the average mass flow rate, and, therefore, the total sample weight. In other words, the sum of the $C_{ij}$ values taken over the duration of the test (T=NΔt=5 s) would be related to either the average mass flow rates, or the total weights of dry wood (*D*) and water (*W*) that moved through the sensor:

(3) $$\begin{array}{c} \sum^{N}\left[\sum_{i=1}^{8}\sum_{j=i+1}^{8}C_{ij}\right]\Delta t=\sum^{N}C\Delta t=\bar{C},\\ =\sum^{N}\left(\alpha +\beta {\dot{m}}_{W}+\gamma {\dot{m}}_{D}\right)\Delta t,\\ =N\alpha \Delta t+\beta \sum^{N}{\bar{\dot{m}}}_{W}\Delta t+\gamma \sum^{N}{\bar{\dot{m}}}_{D}\Delta t,\\ =\alpha^{\prime }+\beta^{\prime }W+\gamma^{\prime }D, \end{array}$$

where *α*, *β*, and *γ* were model coefficients (estimated using regression), relating capacitance *C* and average mass flow rates ${\bar{\dot{m}}}_{W,D}$ of water and wood, respectively.

### 2.3. Experimental Procedures

The general procedure used in these tests was to first calculate a value for total mass *M*, or dry mass *D*, using one of two independent estimation procedures (impact or image), and then use the estimated value in Equation (3), along with the summed capacitance values, $\bar{C}$, to determine the unknown weight, and finally calculate MC.

Tests were conducted using a modified conveyor system to deliver chips into the moisture content sensor. The chips fell off the end of the conveyor, dropped through the capacitance sensor and struck an angled impact plate. Mounted below the plate was a force transducer (DLC101-50, OMEGA Engineering Inc., Norwalk, CT, USA) that provided a voltage output.

The upward-facing surface of the conveyor belt was about 90 cm long by 40 cm wide. For each experimental run, a chip sample was piled to a uniform height on the conveyor belt along its lengthwise centerline within a fixed area, 35 cm in length by 20 cm in width. The belt was then driven, by hand, at a relatively uniform rate (10–20 cm·s${}^{-1}$) to deliver the sample to the sensor system. A data acquisition program (Labview 12.0, National Instruments Co., Austin, TX, USA) collected and recorded capacitance sensor and force transducer output for a fixed time period (5 s), which was sufficient to observe values for the entire sample as it moved through the capacitance sensor past the impact plate. Force data were recorded continuously at 10 kHz. Capacitance observations were recorded at 280 Hz.

### 2.4. Mass Estimation Procedures

#### 2.4.1. Impact Method

Impact forces observed over short periods of time for a stream of particles correlates well with mass flow rate [23]. For these experiments, however, the duration of the impact record was on the order of 5 s and the flow rate of biomass into the sensor could not be controlled. The mass flow rate was, therefore, assumed constant over a fixed time *T* and the quantity to be predicted from the impact data was altered to be total wet mass of each biomass sample, *M*. This single mass value was predicted using analysis of the estimated power spectrum from the complete force record of each sample.

The impact force for individual experiments was sampled at 10 kHz and recorded. A power spectrum estimate for the entire force record was calculated using the Welch method with 2n= 4096-point Hanning window smoothing and overlap of 50%. Power spectrum estimates $s_{k}=s_{0},s_{1},...s_{n}$, for biomass sample number *k* were calculated for the prediction set samples. The spectra were compiled into the set S

$$S={\left[\begin{array}{cc} s_{0} & s_{1}\cdots s_{k}\cdots s_{n} \end{array}\right]}^{T},$$

and the principal component loadings, P, were calculated. The first 50 scores, Q=SP were retained and used in a stepwise regression analysis to predict sample total weight, *M*. The equation used was as follows:

(4) $$M=\beta +\sum_{i=1}^{K}\alpha_{i}p_{i}.$$

The *β* and $\alpha_{i}$ terms were an intercept and slopes for each principal component value, $p_{i}$, estimated using a stepwise least squares technique.

#### 2.4.2. ECT Method

The multi-electrode design of the capacitance sensor enabled calculation, using standard tomographic techniques (ECT), of an image representative of the distribution of dielectric constant within the confines of its sampling region at any point in time. Because wood and water have different dielectric constants than air, it was possible to discriminate the solid phase of material within the sensor and estimate its total area from binary images. If the total area observed in the images could be assumed representative of the solid material quantity within the sensor, then an assumption of uniform density also made it possible to estimate dry wood weight.

A two-step process was used in reconstructing images from the 28 capacitance samples that first applied the linear back projection (LBP) method [24] and, subsequently, used the Landweber iteration algorithm [25] to improve estimates. Both steps required knowledge of the expected distribution of charge density in some reference state, and, for this study, this was taken to be the sensor when filled only with air. This information was encapsulated in a sensitivity matrix, **Ψ**. Sensitivity matrices can be derived from direct measurements, but for this study a simulation approach [26] was used. Simulations were carried out using the ANSYS 13.0 software package (ANSYS Inc., Canonsburg, PA, USA) at 225 nodes established on a triangular grid. This image size was chosen as a reasonable compromise between calculation speed and visual resolution. As implemented, the ECT method was capable of producing 10 permittivity distribution images per second (a single ECT observation consists of 28 capacitance values).

The LBP algorithm can be summarized as below. The measured capacitance C is related to the sensitivity matrix **Ψ**, and the permittivity distribution G through the following relation:

C=ΨG.

We wish to calculate the vector G from the measured capacitance values, so the inverse of the sensitivity matrix is required. In general, **Ψ** is not square and will not possess an inverse, so the transposed sensitivity matrix, $\Psi^{T}$, is used as a starting point in estimating G. Using a pseudo-inverse in the calculation, however, can lead to errors. The Landweber algorithm is meant to minimize those errors by iteratively changing successive estimates of $G_{k+1}$ using the following:

(5) $$G_{k+1}=G_{k}+\alpha \Psi^{T}\left(C-\Psi G_{k}\right).$$

The term *α* was fixed and the algorithm was repeated until the error term $\left|C-\Psi G_{k}\right|$ was less than a predetermined limit.

In this study, there were eight electrodes available for capacitance measurements so there were 28 unique electrode combinations and C was a 28 × 1 vector. The transposed sensitivity matrix $\Psi^{T}$ for this study covered 225 nodes and was therefore a 225 × 28 matrix, each column of which represented the sensitivity simulation result for one electrode combination. The result in applying Equation (5) was a vector G containing 225 gray values representing normalized permittivity at the nodes established for the sensitivity matrix. Those nodes were mapped to a location within the sensor enclosure using their index.

**Figure 2:**
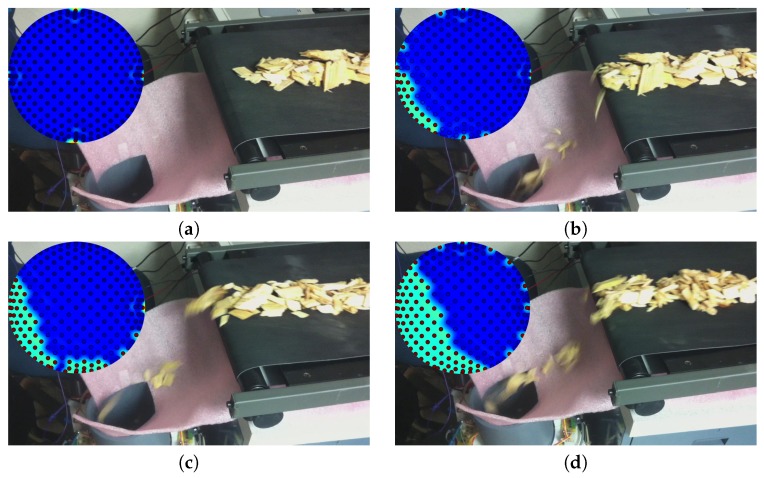
Images of the sensor chip feeding process used in a typical experiment, along with an accompanying image of permittivity distribution. Chips were moved on the conveyor belt and dropped into the sensor opening below the red fabric that was used to capture material falling short. (**a**) illustrated the situation when the sensor was empty; (**b**–**d**) were with increasing quantities of wood chips present in the enclosure. The colored images were interpolated from the estimated *G* matrix using the matplotlib.interpolate function with radial basis kernel.

Figure 2 illustrates how the experiments were conducted and also shows typical images output using the ECT approach. The dark dots in the ECT pictures were the 225 nodes within the enclosure at which the permittivity was estimated. Values at the nodes were used to generate the interpolated images shown in Figure 2. The interpolation was not used in the analysis but did enhance visual interpretation of ECT images.

The dielectric constants of both wood and water are larger than that of air so brightness levels in the images produced using the ECT method should have indicated something about the distribution of those materials. For a particulate material with relatively constant basic shape and uniform dry density, the area occupied by wood and water would be, on average, proportional over time to the mass of dry matter (*D*) currently within the confines of the sensor. These assumptions could be applied in estimating dry matter flow rate through the capacitance sensor by:

- thresholding ECT images to separate biomass from background regions;
- summing the estimated areas over time and correlating the summed areas with measured sample dry mass *D*.

Threshold values to separate biomass from background in ECT images were considered fixed for these experiments. A global threshold was calculated using an experimental procedure in which a single, large wood chip was dropped through the capacitance sensor repeatedly. Every effort was made in dropping the chip to ensure it remained uniformly oriented during its descent and, viewed from above, the visible chip area relative to the total sensor enclosure was fixed. A single wood chip was used to avoid any potential overlap between chips and to maintain a uniform cross-sectional area of flowing material.

The general process in selecting the ECT image threshold was the following:

1. A digital camera installed above the capacitance sensor captured a sequence of grayscale images as the wood chip traveled through the sensing area. These image data were first binarized, then a noise removal process (open/close) employed, to arrive at a threshold gray value resulting in a binary image that closely matched a visual assessment of chip area. Once binarized, the ratio of chip to enclosure area was calculated.
2. The ECT approach was used to simultaneously calculate the dielectric constant distribution as the chip fell through the sensor. This process also resulted in a sequence of grayscale images.
3. A single desktop computer recorded both camera and capacitance sensor data during the chip descent. Image data between the two were matched in time using the CPU clock.
4. The visible binary image of the chip closest in time to the a given ECT image was then selected. A properly thresholded reconstructed permittivity image was assumed to have the same area ratio as that observed in the visible image. The threshold value most closely matching the area ratios in the ECT and visible images was calculated.
5. The global threshold value was taken to be the average of all observed thresholds calculated for images collected during five tests as outlined above.

Due to low resolution of the tomographic reconstruction, the chip boundary for an ECT image was not as accurate as in the visible images and the averaging process was required to ensure that a repeatable threshold was selected. The wood chip volumes determined using the estimated threshold value were closely related to the MC prediction accuracy, and therefore exhibited significant variability. The effect of an inexact threshold, however, would be a bias in estimating a fixed volume and could be corrected using a regression process.

With the camera calibrated ECT image, the dry matter flow rate of wood chips was estimated using the equation below:

(6) $$D=\alpha_{D}+\beta_{D}A,$$

where $\alpha_{D}$ and $\beta_{D}$ were regression coefficients and *A* was observed summed pixels occupied by wood chips. The area ratios were accumulated over the entire run.

All prediction methods (for mass flow and MC) were evaluated using the root mean square error (RMSE) term as defined below.

(7) $$\mathrm{RMSE}=\sqrt{\frac{\sum^{N}{\left(X_{m}-X_{p}\right)}^{2}}{N}}.$$

The RMSE for *N* values was the squared and summed difference between a measured quantity ($X_{m}$) and its prediction based on the sensor outputs, ($X_{p}$). Two forms of RMSE were used, an absolute value as calculated using the above equation, and a relative value that was the RMSE divided by the sample mean, $\bar{X}$.

## 3. Results and Discussion

Calibration of mass flow and MC models for both estimation procedures was done using 60 chip samples selected randomly from those available (79) from a single location (source). Range in total mass and MC of the calibration set was similar to the remaining (49) prediction set samples available from other sources.

### 3.1. Mass Flow Estimation

**Figure 3:**
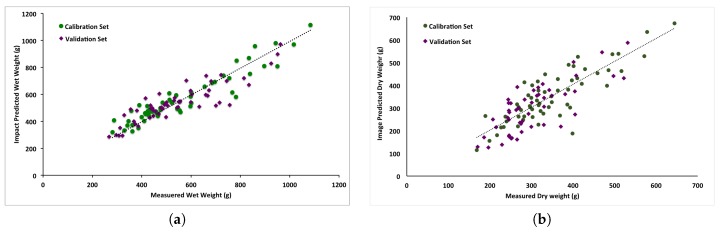
Linear regressions of the predicted total mass flow rate, *M*, using the impact mass flow estimation method (**a**) and dry matter flow rate, *D*, using the ECT method (**b**).

The final regression model relating impact frequency response principal components and total wet mass (*M*, Equation (4)) included five statistically significant principal components (*p* < 0.05) of the 50 tested. The intercept (*β*) was not significantly different from zero (*p* > 0.1), and the overall model was significant (*p* < 0.001) with coefficient of determination ($R^{2}$) for the prediction data set equal to 0.71. The $R^{2}$ for the calibration set was slightly higher, at 0.81. Figure 3a plots the predicted versus measured total wet mass for the calibration and prediction data sets.

Predictions of total dry sample mass *D* using the ECT approach (Equation (6)) were also significant (*p* < 0.001). Figure 3b plots total accumulated biomass area in the ECT images versus measured sample mass for the calibration ($R^{2}=0.71$) and prediction ($R^{2}=0.60$) data sets.

Mass estimates derived using the impact plate approach were less variable than those from the ECT approach. Absolute RMSE of predicted wet mass (*M*) for the impact method was 71.2 g, compared to 69.7 g for dry mass (*D*) estimates from the ECT method. However, because the ECT method predicted dry mass as opposed to wet mass of samples of similar volume, the relative RMSE was higher, and was, in fact, about twice that of the impact method. Table 1 shows a summary of the results.

### 3.2. Moisture Content Estimation

Sample moisture contents were calculated based on total (*M*) and dry matter (*D*) weights using different versions of Equation (3) depending on mass estimation method. Given cumulative capacitance values ($\bar{C}$) and independent weight measures *M* (from the impact method) and *D* (ECT method), Equation (3) was modified into the following two forms.

(8) $$\bar{C}=\alpha^{\prime }+\beta^{\prime }W+\gamma^{\prime }D=\alpha^{\prime }+\beta^{\prime }\left(M-D\right)+\gamma^{\prime }D,$$

(9) $$\mathrm{Impact}\;\mathrm{method}:\;D=\frac{\bar{C}-\alpha_{1}^{\prime }-\beta_{1}^{\prime }M}{\left(\gamma_{1}^{\prime }-\beta_{1}^{\prime }\right)},$$

(10) $$\mathrm{MC}=\frac{M}{D}-1=\frac{-\gamma_{1}^{\prime }M-\bar{C}+\alpha_{1}^{\prime }}{\bar{C}-\alpha_{1}^{\prime }-\beta_{1}^{\prime }M},$$

(11) $$\mathrm{ECT}\;\mathrm{method}:\;M=\frac{\bar{C}-\alpha_{2}^{\prime }-\left(\gamma_{2}^{\prime }-\beta_{2}^{\prime }\right)D}{\beta_{2}^{\prime }},$$

(12) $$\mathrm{MC}=\frac{M}{D}-1=\frac{-\gamma_{2}^{\prime }D+\bar{C}-\alpha_{2}^{\prime }}{\beta_{2}^{\prime }D}.$$

**Figure 4:**
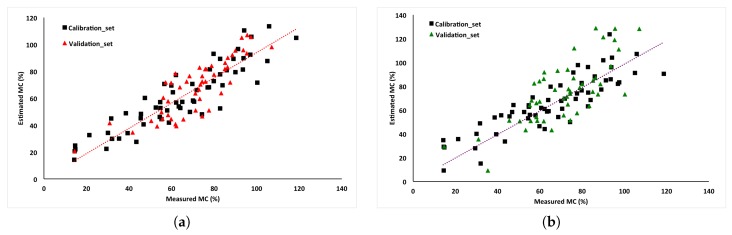
Linear regressions of the true and predicted sample moisture content based on wet mass flow rate (*M*) estimates from (**a**) the impact mass flow method and dry matter flow rate (*D*) estimated using (**b**) the ECT method.

Regression coefficients were estimated using the same calibration samples as in the mass flow experiments (*n* = 60). For this specific system and measurement, $\alpha_{1}^{\prime }=0.982$, $\beta_{1}^{\prime }=1.51\times 10^{-3}$, $\gamma_{1}^{\prime }=8.92\times 10^{-4}$, $\alpha_{2}^{\prime }=1.046$, $\beta_{2}^{\prime }=1.43\times 10^{-3}$, $\gamma_{2}^{\prime }=8.79\times 10^{-4}$. Though the coefficients were from the same equation, their values were slightly different. The difference was mainly from prediction errors of Equations (4) and (6). Predicted moisture contents were shown in Figure 4 as a function of true moisture content determined from oven drying.

A linear regression between estimated and true MC was significant for both mass flow prediction methods (*p* < 0.001), with $R^{2}$ and RMSE values as shown in Table 2. Validation results were also based on the 49 remaining samples used in the mass flow experiments.

### 3.3. Discussion

The basic accuracy of the capacitive sensor in characterizing moisture levels in pine samples was described in earlier work, and it was presumed, since the sensor was not changed, that its contribution to errors in MC estimates in the current study was also the same. The difference in the two mass estimation approaches, therefore, was felt to be the greatest influence on the relative accuracy of the combined sensor in these experiments.

The largest source of error in the impact mass flow assessment was possibly from the use of an angled impact plate as the contact point for the chip flow and the influence it had on the relative energy of chips as they hit. For the conditions in these tests, the difference in kinetic energy of chips impacting the upper versus lower edge of the angled plate was about 14%. In situations where the stream of chips struck the plate consistently off-center, the mass flow estimate would be biased. In the combined sensor as constructed for this study, the two transducers (ECT and impact) were separated physically and correlating the two signals for a specific quantity of chips required the use of a delay. Variations in sample feed rate could have altered the mean delay time and introduced an unknown variation.

The imaging technique used to measure dry matter flow in the ECT approach was limited in that a 2D image was used to estimate sample volume. For a random assortment of orientations of chips within the tube, cross-sectional area might not be uniformly correlated with mass. It was presumed this effect would average out over time, but the assumption was not tested. Inconsistency in placement of chips on the feed conveyor, for example, could have introduced a bias in the results.

Both methods suffered from the slow sampling rate achieved with the ECT data acquisition system. It was possible to generate 10 images per second in real time, but the residence time of a chip within the ECT enclosure as built was about 0.05 seconds. The minimum image generation rate to characterize a variable flow was therefore 20 Hz. Collecting the capacitance data independently from the image processing would have sped the entire process up and been an advantage for the impact method. It should be straightforward, however, to improve the speed of the system in future via hardware and more highly optimized software.

## 4. Conclusions

The objective in presenting the two methods for determining moisture content in this study was to demonstrate an approach combining a capacitance moisture sensor with another measure of sample weight to separate the effect of two mixed phases (dry wood and water) in calculating moisture content of pine chip flows. An impact method was developed to estimate total (wet) weight, and this approach was compared with an alternative method using the tomographic capabilities of the multi-electrode capacitance sensor to calculate dry weight based on an estimate of sample volume. The impact approach resulted in moisture content estimates with RMSE of about 12%, compared to 24% for the volumetric approach. Both approaches were concluded to be feasible for the intended purpose, but the implementation of the ECT method in these tests did not have a sufficient sampling rate to achieve accuracy similar to that of the impact method. In general, the accuracy of the combined impact/capacitance sensor was similar to that of the capacitance sensor alone in predicting MC of fixed samples of known mass. The accuracy of the combined capacitance/volumetric sensor, however, was somewhat less.

## Figures and Tables

**Table 1:** Summary of root mean square error for the two mass flow rate prediction methods.

Method	Sample Set	Mean Sample Weight (g)	Absolute RMSE (g)	Relative RMSE (%)
Impact	Calibration	566.1	58.2	10.3
	Prediction	530.5	71.2	13.4
ECT	Calibration	346.3	62.5	18.1
	Prediction	304.4	69.7	22.9

RMSE: root mean square error. ECT: electrical capacitance tomography.

**Table 2:** Summary of root mean squared error for the two MC prediction methods.

Method	Sample Set	$R^{2}$	Relative RMSE (%)
Impact	Calibration	0.81	11.3
	Prediction	0.71	11.9
ECT	Calibration	0.71	19.1
	Prediction	0.57	24.2

MC: moisture content. RMSE: root mean square error. ECT: electrical capacitance tomography.
